# A synthetic community promotes macadamia growth via root‐microbe interactions and systemic plant responses

**DOI:** 10.1002/imo2.70122

**Published:** 2026-07-09

**Authors:** Xiaofang Yao, Qiumei Liu, Wenlin Wang, Qiujin Tan, Chunheng Zhou, Dejun Li

**Affiliations:** ^1^ Guangxi Key Laboratory of Karst Ecological Processes and Services, Huanjiang Observation and Research Station for Karst Ecosystems, Institute of Subtropical Agriculture Chinese Academy of Sciences Changsha China; ^2^ Genome Analysis Laboratory of the Ministry of Agriculture and Rural Affairs, Agricultural Genomics Institute at Shenzhen Chinese Academy of Agricultural Sciences Shenzhen China; ^3^ Guangxi South Subtropical Agricultural Science Research Institute Guangxi Academy of Agricultural Sciences Nanning Guangxi China

## Abstract

We employed an 18‐month pot experiment and an integrated multi‐omics approach to elucidate the potential mechanisms underlying synthetic community (SynCom)‐mediated macadamia growth promotion. SynCom inoculation enhanced soil nitrogen cycling and potential nutrient (*e.g*., iron) acquisition. It simultaneously increased soil extracellular enzyme activities and enriched beneficial bacteria such as *Rhodanobacter*, *Ferrovibrio*, and *Rhodoblastus* in the root compartment. In addition, SynCom inoculation promoted the accumulation of rhizosphere metabolites, including the microbial siderophore pyochelin, and drove the co‐enrichment of transcriptomic and metabolomic pathways, notably the arginine biosynthesis pathway. This coordinated response may have stimulated nitrogen and iron cycling in the soil, thereby promoting the healthy growth of macadamia.

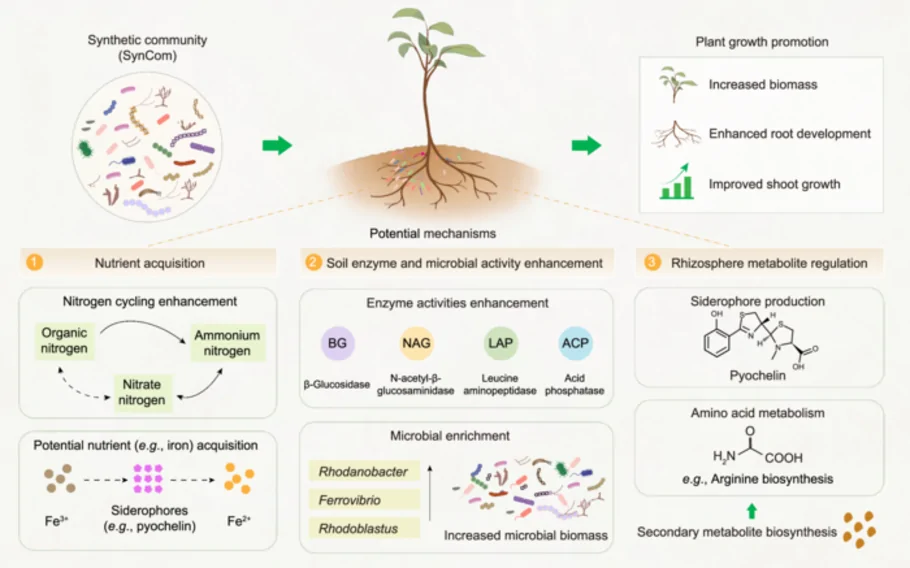

## ETHICS STATEMENT

No animals or humans were involved in this study.

To the Editor,

The plant microbiome plays an important role in plant nutrition, stress tolerance, and pathogen prevention, and is regarded as the second genome of plants [1, 2]. To harness these beneficial functions for sustainable agriculture, the application of synthetic communities (SynComs) has emerged as a promising strategy. SynComs can effectively model or augment the synergistic functions of the natural microbiome, thereby overcoming the ecological inconsistencies and functional limitations typically associated with single‐strain inoculants [3, 4]. However, the complex biological mechanisms underlying prolonged SynCom–host interactions remain incompletely understood.

Current research on SynComs is predominantly confined to short‐lived annual crops, such as rice [5] and cucumber [1]. Consequently, their long‐term stability, potential field applications, and underlying mechanisms in perennial woody crops remain largely unexplored. Emerging evidence from a multi‐omics perspective suggests that introduced beneficial microbes can trigger profound metabolic and transcriptional responses in the host [6]. Nevertheless, it remains unclear whether these induced interactions can be sustained to reshape the root‐associated environment in perennial systems. We hypothesized that SynCom inoculation could induce a coordinated regulation of both the host plant metabolism and the resident rhizosphere microbiome over an extended period.

To address this knowledge gap, we conducted an 18‐month pot experiment using macadamia (*Macadamia integrifolia*) under SynCom inoculation (*Bacillus cereus*, *Bacillus subtilis*, *Pseudomonas fluorescens*, *Enterobacter cloacae*, *Trichoderma harzianum*, *Trichoderma viride*, *Penicillium simplicissimum*, and *Penicillium oxalicum*) and control treatments. Most of these strains exhibit antagonistic activity against the macadamia pathogens (*Pestalotiopsis microspora* and *Cladosporium* sp.; unpublished data) and have been reported as widespread beneficial microbes [7]. Utilizing integrated microbiome, metabolome, and transcriptome analyses, we investigated the long‐term effects of SynCom inoculation on plant‐associated microbial community assembly, soil nutrient profiles, and host metabolic shifts. Our study aims to provide comprehensive, multi‐omics evidence that the SynCom application represents a powerful and sustainable biological strategy to enhance nutrient cycling and promote the fitness of perennial crops.

## 1 EFFECTS OF SYNTHETIC COMMUNITY ON MACADAMIA GROWTH, NUTRIENT CYCLING, AND MICROBIAL COMPOSITION

In the 18‐month pot experiment, SynCom inoculation significantly enhanced macadamia growth parameters, with shoot height, stem diameter, leaf number, shoot dry weight, and root length increasing by 33.4%, 4.0%, 12.6%, 40.3%, and 22.1% (Figure 1A−C). In contrast, no significant differences in most of these parameters were observed between treatments during the first 15 months after inoculation. Additionally, SynCom inoculation reshaped soil nutrient profiles. Soil available nutrient contents were markedly enhanced, with nitrate nitrogen (NO_3_^−^‐N) and dissolved organic nitrogen (DON) increasing by 197.3% and 61.7%, respectively, and microbial biomass nitrogen (MBN) rising by 23.2% (Figure 1D). Correspondingly, the activities of core nutrient‐cycling enzymes, including β‐glucosidase (BG), N‐acetyl‐β‐d‐glucosaminidase (NAG), leucine aminopeptidase (LAP), and acid phosphatase (ACP), increased dramatically, with increases in activity ranging from 63.3% to more than 1200.0% (Figure 1D). These profound enhancements in exoenzyme activities and nitrogen pools suggest that SynCom inoculation effectively stimulates the cycling of nitrogen [8].

**Figure 1:**
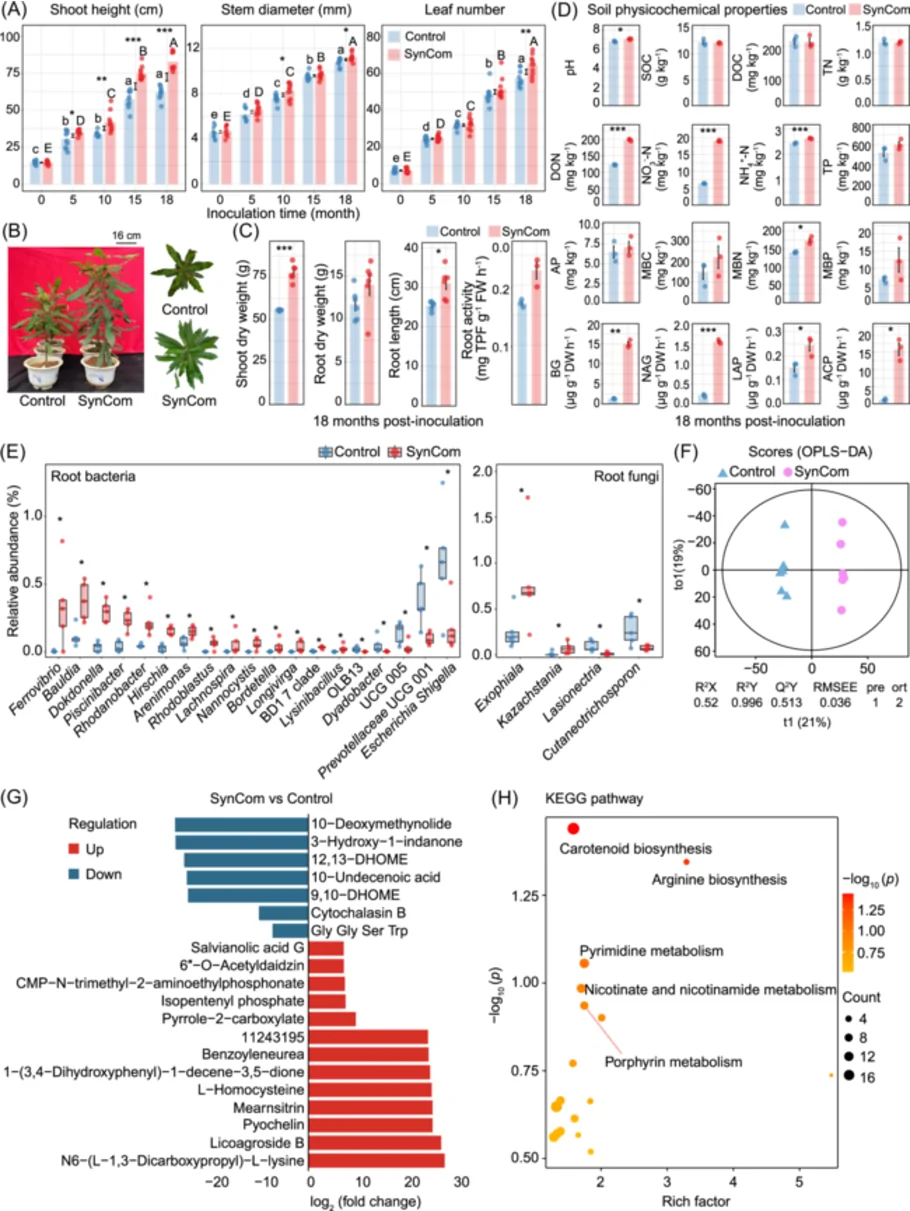
Effects of synthetic community inoculation on macadamia growth, soil physicochemical properties, and rhizosphere metabolome. (A) Dynamic changes in plant growth parameters, including shoot height, stem diameter, and leaf number at different time points following SynCom inoculation. A one‐way ANOVA followed by Tukey's HSD post hoc test was used to evaluate differences among inoculation times within each treatment. Different lowercase letters indicate significant differences among inoculation times within the control treatment, while uppercase letters indicate significant differences within the SynCom treatment (*p* < 0.05). Differences between control and SynCom treatments at each time point were assessed using a two‐sided Student's *t*‐test (*p < 0.05, **p < 0.01, ***p < 0.001). Data are presented as means ± standard error (SE). The same statistical test applies to panels (C−E). (B) Representative images of macadamia seedlings at 18 months post‐inoculation. (C) Comparisons of root growth parameters at 18 months post‐inoculation, including shoot dry weight, root dry weight, root length, and root activity. (D) Comparisons of soil physicochemical properties and enzyme activities, including pH, SOC, DOC, TN, DON, NO_3_^−^‐N, NH_4_^+^‐N, TP, AP, MBC, MBN, MBP, BG, NAG, LAP, and ACP. (E) Differentially abundant bacterial and fungal genera in the root compartment (Relative abundance > 0.1%). Differential taxa were identified using a Wilcoxon rank‐sum test (Mann–Whitney *U* test), and *p*‐values were adjusted using the Benjamini–Hochberg false discovery rate (FDR) correction, and the top 20 genera were selected based on mean abundance differences for visualization. (F) OPLS‐DA score plot showing distinct separation of rhizosphere metabolome between control and SynCom treatments. The model included one predictive component (pre = 1) and two orthogonal components (ort = 2), with R^2^X = 0.52 and R^2^Y = 0.996. The Q^2^Y value was 0.513, and the RMSE was 0.036. SynCom and control samples were separated based on the model output. (G) Top 20 significantly up‐regulated and down‐regulated metabolites. Differential metabolites were identified based on a two‐sided Student's *t*‐test with thresholds of |log_2_ (fold change)| > 1 and *p* < 0.05. (H) KEGG pathway enrichment analysis showing the significantly altered metabolic pathways. Enrichment significance was determined using a hypergeometric test with Benjamini–Hochberg FDR correction. Pathways with FDR < 0.05 were considered significantly enriched. Sample sizes: *n* = 10 for panel (A), *n* = 6 for panel (C, F−H); *n* = 3 for panel (D); *n* = 5 for panel (E). ACP, acid phosphatase; AP, available phosphorus; BG, β‐Glucosidase; DOC, dissolved organic carbon; DW, dry weight; DON, dissolved organic nitrogen; FDR, false discovery rate; FW, fresh weight; KEGG, Kyoto encyclopedia of genes and genomes; LAP, leucine aminopeptidase; MBC, microbial biomass carbon; MBN, microbial biomass nitrogen; MBP, microbial biomass phosphorus; NAG, N‐acetyl‐β‐glucosaminidase; NH_4_^+^‐N, ammonium nitrogen; NO_3_^−^‐N, nitrate nitrogen; SOC, soil organic carbon; TN, total nitrogen; TP, total phosphorus; TPF, triphenyl formazan.

To determine the influence of the SynCom on the plant microbiome assembly, we first explored the effects of spatial compartments on the soil microbiome. Samples were harvested from the bulk soil, rhizosphere, and root compartment following the method of a previous study [9]. SynCom inoculation enriched specific unique taxa without altering overall alpha diversity (Figure S1A−C). This indicates that SynCom inoculation may represent a targeted functional optimization rather than a disruptive ecological shift, a finding consistent with observations from another study [10]. SynCom inoculation reshaped the soil‐root microbiome (Figure S2). In the bulk soil, it suppressed fungi (e.g., *Mortierella*, *Cladosporium*) and promoted bacteria (*Anaeromyxobacter*, *Thiobacillus*, and *Rhodobacter*) involved in iron, sulfur, and nitrogen cycling (Figure S2A,B). In the rhizosphere, fungal suppression persisted, while bacterial enrichment was more selective (*Rhodanobacter*, *Halorubrum*) (Figure S2C,D). The root compartment showed the most pronounced restructuring, with an enrichment of iron‐oxidizing (*Ferrovibrio*), nitrifying/denitrifying (*Rhodoblastus*), and potential growth‐promoting taxa, and the suppression of anaerobic fermenters (*Prevotellaceae*, *Escherichia*, and *Shigella*) (Figure 1E). *Rhodanobacter* exhibited consistent enrichment throughout the soil‐root continuum. Species within this genus, such as *Rhodanobacter thiooxydans* FW510‐R12, are renowned for their denitrification capabilities and stress resilience [11], suggesting their potential contribution as keystone taxa in plant growth and nitrogen turnover.

## 2 EFFECTS OF SYNTHETIC COMMUNITY ON MACADAMIA METABOLOME

To elucidate the mechanisms of SynCom‐induced effects on the macadamia rhizosphere microbial community, we collected macadamia root exudates (which capture a complex of plant‐derived and microbially‐secreted metabolites) following an *in situ* static culture method [12], and analyzed them using ultra‐performance liquid chromatography‐quadrupole time‐of‐flight mass spectrometry (UPLC‐QTOF/MS). A total of 3420 compounds were detected across all samples, resulting in the identification of 647 differential metabolites, with 419 up‐regulated and 228 down‐regulated in the SynCom group (Figure S3). OPLS‐DA revealed distinct metabolite profiles among treatments (Figure 1F). SynCom inoculation significantly upregulated metabolites associated with microbial iron competition, plant secondary metabolism, and amino acid metabolism, including pyochelin, isopentenyl phosphate, l‐Homocysteine, and mearnsitrin. The upregulation of pyochelin (a potent microbial siderophore) provides a mechanistic explanation for the enriched iron‐cycling taxa (e.g., *Ferrovibrio*) and the concomitant suppression of pathogenic fungi via competitive iron exclusion [13, 14]. Conversely, metabolites associated with pathogen activity (cytochalasin B) and lipid peroxidation/oxidative stress (9,10‐DHOME, 12,1_3_^−^DHOME) were downregulated (Figure 1G). Kyoto encyclopedia of genes and genomes (KEGG) analysis further confirmed the metabolic shift towards amino acids, cofactors/vitamins, and terpenoids/polyketides (Figure 1H).

Correlation analysis revealed that the core enriched genera *Rhodanobacter*, *Ferrovibrio*, and *Rhodoblastus* were positively correlated with the upregulated metabolites (N6‐(L‐1,3‐Dicarboxypropyl)‐l‐lysine, pyochelin, isopentenyl phosphate, l‐Homocysteine, and mearnsitrin), but negatively correlated with the downregulated metabolites (12,13‐DHOME, cytochalasin B, and 10‐deoxymethynolide) (Figure S4). These upregulated metabolites were primarily involved in lysine biosynthesis and degradation, amino acid biosynthesis, sulfur metabolism, and secondary metabolite biosynthesis, whereas 12,13‐DHOME was mainly associated with linoleic acid metabolism. Crucially, both the enriched bacterial taxa (e.g., *Rhodanobacter*, *Rhodoblastus*) and their associated upregulated metabolites (e.g., pyochelin, N6‐(L‐1,3‐Dicarboxypropyl)‐l‐lysine) exhibited strong positive correlations with soil nitrogen‐cycling parameters (MBN, DON, NO_3_^−^‐N, NH_4_^+^‐N, and related enzymes) and plant growth parameters (root length, shoot biomass). In contrast, downregulated metabolites (e.g., 12,13‐DHOME, cytochalasin B) were significantly negatively associated with soil nitrogen availability and plant growth (Figure S5A−D). These findings suggest that shifts in the microbial community are closely associated with changes in both soil nutrient turnover and nitrogen distribution within the plant [15, 16].

## 3 MULTI‐OMICS REVEALS SYNCOM‐MEDIATED REGULATION OF HOST METABOLISM AND TRANSCRIPTION

To elucidate the mechanisms underlying SynCom‐induced changes in microbial community structure and rhizosphere metabolite profiles, we next performed integrated transcriptomic and metabolomic analyses. Two‐way orthogonal partial least squares (O2PLS) analysis showed a strong correlation between the root exudate metabolome and the transcriptome, suggesting that specific genes and metabolites are tightly linked (Figure 2A). The nine‐quadrant diagram further illustrates that the majority of differentially expressed genes (DEGs) and differential metabolites are co‐localized in the co‐upregulation (top‐right) and co‐downregulation (bottom‐left) quadrants (Figure 2B), demonstrating a strong positive correlation between DEGs and metabolic fluxes. KEGG analysis identified 43 pathways significantly co‐enriched by both DEGs and differential metabolites (Figure 2C), which were primarily associated with amino acid metabolism, secondary metabolite biosynthesis, and lipid metabolism, among which, the arginine biosynthesis pathway was notably co‐enriched (Figure 2D). The enrichment of arginine may accelerate the absorption and transport of NH_4_^+^‐N, thereby improving plant stress tolerance [17]. Furthermore, the co‐enrichment of the siderophore pyochelin and the iron‐cycling bacterium *Ferrovibrio* indicates a potential role for SynCom inoculation in facilitating plant iron acquisition [14, 18].

**Figure 2:**
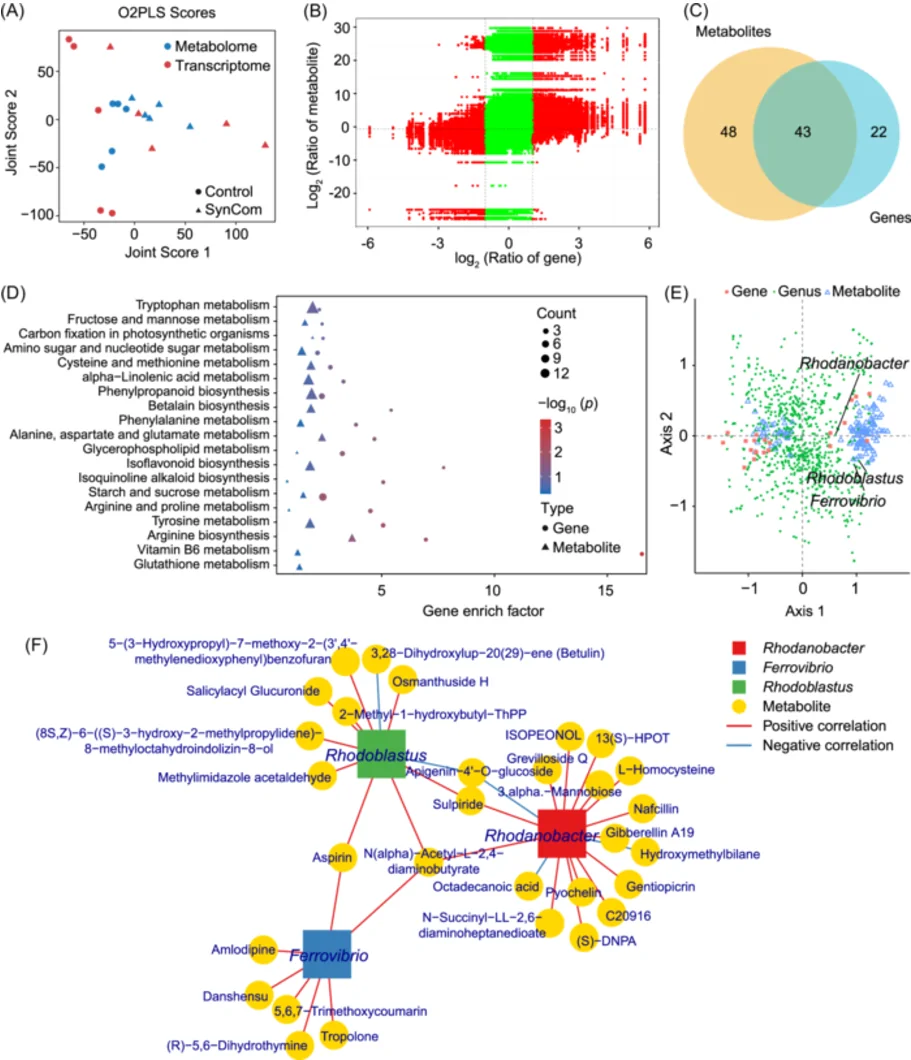
Integrated analysis of microbiome, metabolome, and transcriptome. (A) O2PLS joint score plot based on metabolome and transcriptome data. (B) Nine‐quadrant plot of correlations between genes and metabolites. Gene and metabolite abundance data were first standardized using *z*‐score transformation. Pearson correlation analysis was then performed across all gene‐metabolite pairs within each treatment. Significant correlations were filtered using thresholds of |correlation coefficient (CC)| > 0.80 and *p* < 0.05. Red and green dots indicate significantly positive and negative correlations, respectively. (C) Venn diagram showing pathway overlaps between metabolome and transcriptome. (D) KEGG pathway enrichment analysis identifying pathways co‐enriched with both DEGs and differential metabolites. Circles and triangles denote genes and metabolites, respectively. (E) Multiple co‐inertia analysis showing the spatial distribution of microbial genera, metabolites, and genes. Red squares, green circles, and open triangles denote genes, genera, and metabolites, respectively. (F) Co‐occurrence network analysis between key bacterial genera and differential metabolites. Square nodes represent the three prominent beneficial bacterial genera enriched by SynCom. Yellow circular nodes represent highly correlated differential metabolites. Red edges indicate significant positive correlations, while blue edges indicate significant negative correlations. DEGs, differentially expressed genes; KEGG, Kyoto encyclopedia of genes and genomes; O2PLS, two‐way orthogonal partial least squares.

Finally, co‐inertia analysis visually confirmed this synergy. The ordination triplot revealed that key SynCom‐enriched genera (*Rhodanobacter*, *Rhodoblastus*, and *Ferrovibrio*) were spatially co‐located with dense clusters of DEGs and upregulated metabolites along Axis 1, mathematically signifying a positive coupling (Figure 2E). The subsequent correlation network highlighted *Rhodanobacter* as a central hub, densely connected to pyochelin and l‐Homocysteine. Furthermore, metabolites such as N(alpha)‐acetyl‐L‐2,4‐diaminobutyrate served as bridging nodes connecting different bacterial genera (Figure 2F). These shared metabolic hubs suggest strong metabolic interactions among root‐associated microorganisms, potentially stabilizing the microbial community and facilitating long‐term plant growth promotion.

In summary, unlike most previous SynCom studies conducted in annual crops over relatively short time scales, our findings suggest that SynCom‐induced reprogramming of host metabolism and microbiome assembly may persist in a perennial woody crop system, highlighting the potential importance of long‐term host–microbe feedbacks in SynCom performance. The SynCom may function as an ecological catalyst that facilitates coordinated interactions between the host plant and the rhizosphere microbiome. Through specific transcriptional regulation, the host may reshape its root exudate profile, which in turn could contribute to a rhizosphere environment that promotes the accumulation of microbial functional metabolites, including the siderophore pyochelin. The resulting rhizosphere metabolic environment may selectively enrich and stabilize key functional taxa, such as *Rhodanobacter*, *Ferrovibrio*, and *Rhodoblastus*, facilitate microbial metabolic interactions, and potentially contribute to the cycling and transformation of essential nutrients, particularly nitrogen. Ultimately, the coordinated interplay among host transcriptional regulation, root exudate remodeling, and microbial community assembly may be associated with improved nutrient acquisition and plant growth performance. Given the inherent limitations of studying a perennial woody crop system, our conclusions are based on a systems biology‐derived ecological metabolic model inferred from multi‐omics associations. Advanced sequencing technologies, in particular, served as the foundational tool enabling these multi‐omics insights [19]. Future studies employing reverse‐genetics approaches and targeted functional experiments will be required to validate the causal relationships proposed here. Collectively, our findings provide a theoretical basis for understanding the potential mechanisms by which SynCom inoculation may influence nutrient cycling and support the growth and fitness of perennial crops.

## AUTHOR CONTRIBUTIONS

**Xiaofang Yao**: Methodology; software; data curation; investigation; visualization; formal analysis; writing—original draft; writing—review and editing. **Qiumei Liu**: Investigation; supervision; project administration; writing—review and editing. **Wenlin Wang**: Validation; funding acquisition; writing—review and editing. **Qiujin Tan**: Investigation; validation; writing—review and editing. **Chunheng Zhou**: Validation; writing—review and editing. **Dejun Li**: Conceptualization; methodology; data curation; validation; supervision; funding acquisition; project administration; resources; writing—review and editing.

## CONFLICT OF INTEREST STATEMENT

The authors declare no conflicts of interest.

## AI STATEMENT

The initial draft of this manuscript was polished for grammar and sentence structure using ChatGPT (GPT‐5.5; OpenAI, San Francisco, CA, USA). All AI‐generated content was meticulously reviewed and edited by the authors to ensure factual accuracy and prevent any potential AI‐induced bias.

## Supporting information


**Figure S1:** Effects of a synthetic community (SynCom) inoculation on microbial community structure in bulk soil, rhizosphere, and root compartment.
**Figure S2:** Differentially abundant bacterial and fungal genera in bulk soil and rhizosphere under SynCom and control treatments.
**Figure S3:** Volcano plot of differential metabolites between the SynCom and control treatments.
**Figure S4:** Correlations between differential metabolites and differential root bacterial genera.
**Figure S5:** Correlations among differential metabolites, differential root bacterial genera, plant growth parameters, and soil properties.

**Table S1:** Basic physicochemical properties of the experimental soil.

## Data Availability

The data that support the findings of this study are openly available in Genome Sequence Archive at https://ngdc.cncb.ac.cn/gsa, reference numbers CRA029957, CRA030019, OMIX011852. The raw sequence data used in this study are available in the Genome Sequence Archive under the accession numbers, including amplified sequencing (CRA029957, https://ngdc.cncb.ac.cn/gsa/search?searchTerm=CRA029957), Transcriptome (CRA030019, https://ngdc.cncb.ac.cn/gsa/search?searchTerm=CRA030019), and Metabolome (OMIX011852, https://ngdc.cncb.ac.cn/omix/release/OMIX011852). The data and scripts used are saved in GitHub (https://github.com/yaoxiaofang1/Macadamia-growth-promotion). Supplementary materials (methods, figures, table, graphical abstract, slides, videos, Chinese translated version, and updated materials) may be found in the online DOI or iMeta Science http://www.imeta.science/imetaomics.
