# Supplementary material for: A synthetic community promotes macadamia growth via root‐microbe interactions and systemic plant responses
Source: IMetaOmics. 2026 Jul 9;3(3):e70122. doi: 10.1002/imo2.70122 (PMC13674419; doi:10.1002/imo2.70122)
Supplement: Supplementary file 1 [file IMO2-3-e70122-s002.docx]

Supporting information to

**A synthetic community promotes macadamia growth via root-microbe interactions and systemic plant responses**

**Running title**: Macadamia growth promotion by a synthetic community

Xiaofang Yao^1, 2^, Qiumei Liu^1^, Wenlin Wang^3^, Qiujin Tan^3^, Chunheng Zhou^3^, Dejun Li^1*^

^1^Guangxi Key Laboratory of Karst Ecological Processes and Services, Huanjiang Observation and Research Station for Karst Ecosystems, Institute of Subtropical Agriculture, Chinese Academy of Sciences, Changsha 410125, China

^2^Genome Analysis Laboratory of the Ministry of Agriculture and Rural Affairs, Agricultural Genomics Institute at Shenzhen, Chinese Academy of Agricultural Sciences, Shenzhen 518120, China

^3^Guangxi South Subtropical Agricultural Science Research Institute, Guangxi Academy of Agricultural Sciences, Guangxi 530007, China

*Correspondence: dejunli@isa.ac.cn (Dejun Li)

**METHODS**

**Antagonistic strain screening and synthetic community construction**

The microbial strains used to construct the SynCom were isolated from the rhizosphere soil of an organic vegetable farm (36°00'26" N, 116°52'33" E) in Feicheng City, Shandong Province, China, which has a 30-year history of organic fertilization. We intentionally sourced strains from a non-macadamia habitat to capture functionally diverse and environmentally robust microorganisms, aiming to construct a SynCom with broad functional applicability rather than strict host specificity. For fungal isolation, a soil suspension was serially diluted, and 0.1 mL of the 10^-3^, 10^-4^, and 10^-5^ dilutions were plated in triplicate onto potato dextrose agar and incubated at 28°C for 3–10 days. For bacterial isolation, 0.1 mL of the 10^-5^, 10^-6^, and 10^-7^ dilutions were plated in triplicate onto Luria-Bertani agar (Tryptone 10 g, Yeast Extract 5 g, NaCl 10 g, Agar 15 g, per 1 L, pH 7.0) and incubated at 37°C for 3–10 days [1]. Pure isolates were subsequently screened for their antagonistic activity against macadamia pathogens (*Pestalotiopsis microspora* and *Cladosporium* sp.) using a standard dual-culture assay on potato dextrose agar plates. The pairwise compatibility tests were performed among the shortlisted antagonistic bacterial and fungal strains to ensure their ability to coexist without significant mutual inhibition. Genomic DNA was extracted from the most promising antagonistic strains using the PowerSoil DNA Isolation Kit (MoBio Laboratories, Carlsbad, CA, USA). Strains were identified by sequencing the 16S rRNA gene (for bacteria) or the Internal Transcribed Spacer (ITS) region (for fungi).

Based on phylogenetic diversity and functional potential, strain selection followed a function-oriented framework integrating (i) antagonistic activity against macadamia decline-associated pathogens; (ii) compatibility among candidate strains; and (iii) complementary ecological functions reported in the literature, including nitrogen fixation, phosphate solubilization, cellulolytic activity, siderophore production, and pathogen suppression [2,3]. Finally, 8 strains were selected to construct a SynCom, including four bacterial strains (*Bacillus cereus*, *Bacillus subtilis*, *Pseudomonas fluorescens*, and *Enterobacter cloacae*) and four fungal strains (*Trichoderma harzianum*, *Trichoderma viride*, *Penicillium simplicissimum*, and *Penicillium oxalicum*). These strains were chosen to represent complementary ecological functions in nutrient cycling, organic matter decomposition, and broad-spectrum antagonism against pathogens [4,5].

**Pot experiment design**

The pot experiment was conducted in a nursery greenhouse at the Guangxi Subtropical Agriculture Research Institute in Longzhou, Chongzuo, Guangxi, China. Six-month-old macadamia seedlings of uniform size were used. The basic physicochemical properties of the soil are detailed in Table S1. The experiment consisted of two treatments: a control group and a SynCom-inoculated group (SynCom). Each treatment included 30 replicate plants (one seedling per pot). Each pot (32 cm diameter) contained 6 kg of fresh soil amended with 0.8% (w/w) organic fertilizer (commercial bio-organic fertilizer, ≥ 40.0% organic matter, ≥ 2.0 × 10^8^ CFU g^-1^ viable microorganisms).

After a one-week acclimation period, seedlings in the SynCom group were inoculated the SynCom suspension (bacterial and fungal components adjusted to 1 × 10^7^ CFU·mL^-1^ and 1 × 10^7^ spores mL^-1^, respectively, resulting in approximately 1 × 10^6^ CFU g^-1^ or spores g^-1^ soil per strain). This inoculation level falls within the range commonly used in SynCom and plant growth-promoting microorganism studies and has been shown to support effective root colonization and establishment of introduced [2,6,7]. Control plants were given an equal amount of sterile Luria-Bertani and potato dextrose broth. Plants were watered daily, and the experiment was conducted for 18 months.

Plant growth parameters, including shoot height (from the soil surface to the apical meristem), stem diameter (measured 10 cm above the soil surface), and leaf number, were recorded at 0, 5, 10, 15, and 18 months after inoculation. After 18 months, fresh biomass of the shoots and roots was determined. The samples were then oven-dried at 80°C for 72 h to obtain the dry biomass [8]. Five biological replicates were randomly selected from each treatment for destructive sampling. Soil, rhizosphere, and root samples were collected from each plant. Bulk soil was defined as the non-rhizosphere soil collected from the pot at locations distant from the root system, after discarding the top 2 cm layer. To collect rhizosphere samples, seedlings were carefully removed from the pots, and loosely adhering soil was gently shaken off. The entire root system was then placed in a 50 mL centrifuge tube containing 25 mL of sterile PBS. The tube was shaken at 180 rpm for 20 min. The roots were then transferred to a new tube. The resulting soil suspension was centrifuged at 4000 × g for 20 min at 16°C, and the pellet was collected as the rhizosphere soil [9]. For root samples, the washed roots were further sonicated in fresh PBS (40 Hz, 30 s, twice) to remove residual soil particles adhering to the root surface. All samples (bulk soil, rhizosphere, and roots) were immediately flash-frozen in liquid nitrogen and stored at –80°C for downstream analyses [10].

Root vitality was measured based on dehydrogenase activity using the 2,3,5-triphenyltetrazolium chloride reduction method with a commercial kit (R30350-50T, Yuanye Bio, Shanghai, China) following the manufacturer’s protocol [11]. The amount of triphenyl formazan produced was quantified spectrophotometrically at 485 nm. Root vitality was expressed as mg triphenyl formazan g^-1^ fresh weight h^-1^.

Soil pH was measured in a 1:2.5 soil-to-water slurry. Soil water content (SWC) was determined gravimetrically. Total carbon (TC) and total nitrogen (TN) were measured using an elemental analyzer (Elementar Vario EL cube, Germany). Soil organic carbon (SOC) was determined by the potassium dichromate oxidation method. Available phosphorus (AP) was extracted with 0.5 mol L^-1^ NaHCO_3_. Nitrate nitrogen (NO_3_^-^-N), ammonium nitrogen (NH_4_^+^-N), Dissolved organic carbon (DOC), and dissolved organic nitrogen (DON) were extracted with 0.05 mol L^-1^ K_2_SO_4_. DOC was determined using a wet-oxidation method, total dissolved nitrogen (TDN) was measured from the same K_2_SO_4_ extracts after alkaline potassium persulfate digestion (0.15 mol L^-1^ NaOH and 3% K_2_S_2_O_8_), and DON was calculated by subtracting mineral nitrogen content from TDN [12]. All dissolved nutrients were quantified using an AutoAnalyzer 3 continuous flow analyzer (SEAL Analytical, Germany) [13].

Microbial biomass carbon (MBC), microbial biomass nitrogen (MBN), and microbial biomass phosphorus (MBP) were determined using the chloroform fumigation-extraction method. MBC and MBN were extracted with 0.05 mol L^-1^ K_2_SO_4_ and calculated using conversion factors (kEC = 0.45, kEN = 0.54). MBP was extracted with 0.5 mol L^-1^ NaHCO_3_ (pH 8.5) and calculated with a conversion factor of kEP = 0.40.

The activities of four key extracellular enzymes involved in carbon, nitrogen, and phosphorus cycling, namely β-Glucosidase (BG), N-acetyl-β-glucosaminidase (NAG), leucine aminopeptidase (LAP), and acid phosphatase (ACP), were assayed using a microplate fluorometric method with 4-methylumbelliferone (MUB)-linked substrates [13]. Enzyme activities were expressed as nmol MUB released per hour per gram of dry soil (nmol h^-1^ g^-1^).

Root exudates were collected *in situ*. The root systems of intact plants were gently washed, and a portion was enclosed in filter paper moistened with deionized water. The assembly was placed in a 50 mL centrifuge tube, which was then re-buried in the original soil for 24 h. After retrieval, the roots were immersed in 100 mL of deionized water and shaken for 30 min. The resulting solution containing the exudates was collected, concentrated by rotary evaporation, and lyophilized [14]. This *in situ* method was chosen to minimize root disturbance and capture exudate profiles more representative of the natural state, although minor procedural stress cannot be entirely excluded. The dried exudate powder was stored at –80°C for downstream analyses.

**Multi-omics analyses**

Multi-omics analyses were performed on 18-month post-inoculation samples. Amplicon sequencing (bulk soil, rhizosphere, and root) and transcriptomic analyses used five biological replicates per treatment (*n* = 5). Metabolomics of root exudates used six replicates per treatment (*n* = 6). All samples were derived from independent plants.

Total genomic DNA was extracted from 0.25 g of bulk soil, rhizosphere, and root samples using the DNeasy PowerSoil Pro Kit (Qiagen, Germany). For bacterial community profiling, the V3–V4 region of the 16S rRNA gene was amplified using primers 338F/806R for soil samples and 335F/769R for root samples. For fungi, the ITS1 region of the rRNA gene was amplified using primers ITS1F/ITS1R for all samples. PCR amplicons were purified, quantified, and pooled. Sequencing was performed on an Illumina NovaSeq 6000 platform (PE250) at Biomarker Technologies (Beijing, China). Raw sequencing reads were processed using QIIME 2 (v2023.2). DADA2 was used to denoise, merge, and generate amplicon sequence variants (ASVs) [15]. Taxonomy was assigned using the SILVA database (v138.1) for bacteria and the UNITE database (v8.3) for fungi.

Total RNA was extracted from root tissues using the RNeasy Plant Mini Kit (Qiagen). RNA quality and quantity were assessed, and sequencing libraries were prepared. Paired-end (150 bp) sequencing was performed on an Illumina NovaSeq 6000 platform. Raw reads were filtered to obtain high-quality clean reads, which were subsequently mapped to the macadamia reference genome using HISAT2 [16]. Transcript assembly and gene-level read counts were generated [17]. Differential expression analysis was performed using DESeq2 based on raw count matrices.

The dried exudate powder was analyzed by non-targeted metabolomics using an ultra-high-performance liquid chromatography-quadrupole time-of-flight mass spectrometry (UPLC-QTOF/MS) platform (Waters Acquity I-Class PLUS coupled to a Xevo G2-XS QTOF) at Biomarker Technologies (Beijing, China). Raw data were processed using Progenesis QI software for peak alignment, normalization, and metabolite identification against public databases.

**Statistical analysis**

Statistical analyses were performed in R (v4.4.1). Normality of data was assessed using the Shapiro-Wilk test. Independent samples *t*-test or Mann-Whitney *U* test was used for two-group comparisons. One-way ANOVA followed by Tukey’s HSD post-hoc tests was used for multi-group comparisons. Differential metabolites were identified based on two-sided Student’s *t*-test with thresholds of |log_2_ (fold change)| > 1 and *p* < 0.05 [18,19]. Microbiome alpha diversity was assessed using the Shannon index, and beta diversity was analyzed using Principal Coordinates Analysis (PCoA) on Bray-Curtis dissimilarity matrices, with statistical significance determined by PERMANOVA. Co-occurrence networks were constructed based on Spearman correlations. Multi-omics integration was performed using two-way orthogonal partial least squares (O2PLS) and multiple co-inertia analysis. All data are presented as means ± standard error (SE). Significance levels are indicated in figure legends.

**REFERENCES**

1.	Jeff-Ego, Olumide S., Olufemi A. Akinsanmi. 2019. “Botryosphaeriaceae causing branch dieback and tree death of macadamia in Australia.” *Australasian Plant Pathology* 48: 59–64. https://doi.org/10.1007/s13313-018-0604-6

2.	Niu, Ben, Joseph Nathaniel Paulson, Xiaoqi Zheng, Roberto Kolter. 2017. “Simplified and representative bacterial community of maize roots.” *Proceedings of the National Academy of Sciences of the United States of America* 114: E2450–E2459. https://doi.org/10.1073/pnas.1616148114

3.	Vorholt, Julia A., Christine Vogel, Charlotte I. Carlström, Daniel B. Müller. 2017. “Establishing causality: Opportunities of synthetic communities for plant microbiome research.” *Cell Host & Microbe* 22: 142–155. https://doi.org/10.1016/j.chom.2017.07.004

4.	Zhou, Hu, Zuo-hua Ren, Xue Zu, Xi-yue Yu, Hua-jun Zhu, Xiao-juan Li, Jie Zhong, Er-ming Liu. 2021. “Efficacy of plant growth-promoting bacteria *Bacillus cereus* YN917 for biocontrol of rice blast.” *Frontiers in Microbiology* 12: 684888. https://doi.org/10.3389/fmicb.2021.684888

5.	Molina-Santiago, Carlos, John R. Pearson, Yurena Navarro, María Victoria Berlanga-Clavero, Andrés Mauricio Caraballo-Rodriguez, Daniel Petras, María Luisa García-Martín, et al. 2019. “The extracellular matrix protects *Bacillus subtilis* colonies from *Pseudomonas* invasion and modulates plant co-colonization.” *Nature Communications* 10: 1919. https://doi.org/10.1038/s41467-019-09944-x

6.	Liu, Hongwei, Zhiguang Qiu, Jun Ye, Jay Prakash Verma, Jiayu Li, Brajesh K. Singh. 2022. “Effective colonisation by a bacterial synthetic community promotes plant growth and alters soil microbial community.” *Journal of Sustainable Agriculture and Environment* 1: 30–42. https://doi.org/10.1002/sae2.12008

7.	Tsolakidou, Maria-Dimitra, Ioannis A. Stringlis, Natalia Fanega-Sleziak, Stella Papageorgiou, Antria Tsalakou, Iakovos S. Pantelides. 2019. “Rhizosphere-enriched microbes as a pool to design synthetic communities for reproducible beneficial outputs.” *FEMS Microbiology Ecology* 95: fiz138. https://doi.org/10.1093/femsec/fiz138

8.	Cheng, Xia, Rong Wang, Xingzhu Liu, Lijuan Zhou, Minghua Dong, Muzammal Rehman, Shah Fahad, Lijun Liu, Gang Deng. 2022. “Effects of light spectra on morphology, gaseous exchange, and antioxidant capacity of industrial hemp.” *Frontiers in Plant Science* 13: 937436. https://doi.org/10.3389/fpls.2022.937436

9.	Wang, Zhenghong, Zewen Li, Yujie Zhang, Jingye Liao, Kaixiang Guan, Jingxuan Zhai, Pengfei Meng, et al. 2024. “Root hair developmental regulators orchestrate drought triggered microbiome changes and the interaction with beneficial Rhizobiaceae.” *Nature Communications* 15: 10068. https://doi.org/10.1038/s41467-024-54417-5

10.	Wei, Zhong, Yian Gu, Ville-Petri Friman, George A. Kowalchuk, Yangchun Xu, Qirong Shen, Alexandre Jousset. 2019. “Initial soil microbiome composition and functioning predetermine future plant health.” *Science Advances* 5: eaaw0759. https://doi.org/10.1126/sciadv.aaw0759

11.	Dong, Youming, Minling Gao, Zhengguo Song, Weiwen Qiu. 2020. “Microplastic particles increase arsenic toxicity to rice seedlings.” *Environmental Pollution* 259: 113892. <https://doi.org/10.1016/j.envpol.2019.113892>

12.	Yang, Xinyi, Pengpeng Duan, Andrew T. Nottingham, Wolfgang Wanek, Lettice C. Hicks, Luiz A. Domeignoz-Horta, Peilei Hu, et al. 2026. “Land use overrides climatic controls on soil organic nitrogen transformations: Contrasting responsiveness between forest and cropland ecosystems.” *Functional Ecology* 00: 1–15. https://doi.org/10.1111/1365-2435.70366

13.	Yao, Xiaofang, Qiumei Liu, Dejun Li. 2024. “Mechanism underlying effects of cellulose-degrading microbial inoculation on amino acid degradation and biosynthesis during composting.” *Bioresource Technology* 403: 130899. <https://doi.org/10.1016/j.biortech.2024.130899>

14.	Oburger, Eva, David L. Jones. 2018. “Sampling root exudates - Mission impossible?” *Rhizosphere* 6: 116–133. https://doi.org/10.1016/j.rhisph.2018.06.004

15.	Gao, Yunyun, Kai Peng, Defeng Bai, Xiao-Ye Bai, Yujing Bi, Anqi Chen, Baodong Chen, et al. 2024. “The Microbiome Protocols eBook initiative: Building a bridge to microbiome research.” *iMeta* 3: e182. https://doi.org/10.1002/imt2.182

16.	Kim, Daehwan, Ben Langmead, Steven L. Salzberg. 2015. “HISAT: a fast spliced aligner with low memory requirements.” *Nature Methods* 12: 357–360. https://doi.org/10.1038/nmeth.3317

17.	Pertea, Mihaela, Geo M. Pertea, Corina M. Antonescu, Tsung-Cheng Chang, Joshua T. Mendell, Steven L. Salzberg. 2015. “StringTie enables improved reconstruction of a transcriptome from RNA-seq reads.” *Nature Biotechnology* 33: 290–295. https://doi.org/10.1038/nbt.3122

18.	Wold, Svante, Michael Sjöström, Lennart Eriksson. 2001. “PLS-regression: a basic tool of chemometrics.” *Chemometrics and Intelligent Laboratory Systems* 58: 109–130. https://doi.org/10.1016/S0169-7439(01)00155-1

19.	Xia, Jianguo, David S. Wishart. 2016. “Using MetaboAnalyst 3.0 for comprehensive metabolomics data analysis.” *Current Protocols in Bioinformatics* 55: 14.10.11–14.10.91. https://doi.org/10.1002/cpbi.11

**Figure S1 Effects of a synthetic community (SynCom) inoculation on microbial community structure in bulk soil, rhizosphere, and root compartment.** (A) Venn diagrams showing the distribution of shared and unique bacterial and fungal amplicon sequence variants (ASVs) between SynCom and control treatments. (B) Alpha diversity comparison based on the Shannon index. (C) Beta diversity analysis using principal coordinates analysis (PCoA) based on Bray-Curtis dissimilarity.

**Figure S2 Differentially abundant bacterial and fungi genera in bulk soil and rhizosphere under SynCom and control treatments.** (A and B) Bacterial (A) and fungal (B) genera in the bulk soil. (C and D) Bacterial (C) and fungal (D) genera in the rhizosphere. Statistical differences between control and SynCom treatments were assessed using the two-sided Wilcoxon rank-sum test. *p*-values were adjusted using the Benjamini-Hochberg method. Asterisks denote significant differences between the control and SynCom treatments (**p* < 0.05, ***p* < 0.01, ****p* < 0.001).

**Figure S3 Volcano plot of differential metabolites between the SynCom and control treatments.** A total of 419 metabolites were significantly upregulated (*e.g.*, Pyochelin and L-Homocysteine) and 228 were significantly downregulated.

**Figure S4 Correlations between differential metabolites and differential root bacterial genera.** Spearman’s rank correlation test was performed. Significance levels are denoted as: **p* < 0.05, ***p* < 0.01, ****p* < 0.001.

**Figure S5 Correlations among differential metabolites, differential root bacterial genera, plant growth parameters, and soil properties.** (A) Correlations between differential metabolites and plant growth parameters. (B) Correlations between differential metabolites and soil properties. (C) Correlations between differential root bacterial genera and plant growth parameters. (D) Correlations between differential root bacterial genera and soil properties. Spearman’s rank correlation test was performed. Significance levels are denoted as: **p* < 0.05, ***p* < 0.01, and ****p* < 0.001. Data are presented as means ± standard errors (*n* = 3). Abbreviation: DON, Dissolved organic nitrogen; NO_3_^-^-N, Nitrate nitrogen; NH_4_^+^-N, Ammonium nitrogen; MBN, Microbial biomass nitrogen; MBP, Microbial biomass phosphorus; BG, β-Glucosidase; NAG, N-acetyl-β-glucosaminidase; LAP, Leucine aminopeptidase; ACP, Acid phosphatase.
